# Complete Genome Sequences of Three phi29-Like *Bacillus cereus* Group *Podoviridae*

**DOI:** 10.1128/genomeA.00701-17

**Published:** 2017-07-20

**Authors:** Ivan Erill, Steven M. Caruso

**Affiliations:** Department of Biological Sciences, University of Maryland, Baltimore County, Baltimore, Maryland, USA

## Abstract

Three double-stranded DNA phi29-like *Bacillus cereus* group bacteriophages, BeachBum, Harambe, and SerPounce, were identified and characterized. BeachBum and Harambe are closely related but are remarkably different from previously identified phi29-like phages. SerPounce is substantially closer to other phi29-like phages, enabling the identification of its prohead RNA (pRNA) gene.

## GENOME ANNOUNCEMENT

Three phi29-like *Podoviridae* (1) were isolated from soil samples at different sites in Maryland and sequenced and characterized by UMBC Phage Hunters as part of the 2016 to 2017 SEA-PHAGES course (2) using *Bacillus thuringiensis* subsp. *kurstaki* (ATCC 33679) as the host: *Bacillus* phages BeachBum, Harambe, and SerPounce. *B. thuringiensis* subsp. *kurstaki* is a member of the *Bacillus cereus* group of closely related, Gram-positive, spore-forming, aerobic, rod-shaped species (3). All three phages have linear double-stranded DNA (dsDNA) genomes ending in short inverted repeats of 8 to 16 bp. Protein-coding-gene start codons consisted of 87.9% AUG, 5.6% GUG, and 6.5% UUG among the three phages. Additional information about these and other *Bacillus* phages isolated by undergraduate researchers can be found on the *Bacillus* phages database (http://bacillus.phagesdb.org/).

BeachBum, the smallest of the three phages, has a 21,054-bp-long genome, with 30 protein-coding genes and a G+C content of 35.4%. Harambe’s genome is 21,684 bp in length and contains 33 protein-coding genes, 30 of which are also found in BeachBum, and a G+C content of 35.2%. BeachBum and Harambe are very similar phages, with an average nucleotide identity (ANI) of 96% (4); however, they are quite different from previously identified phi29-like phages, displaying ANIs of 60% or below. SerPounce has a 27,206-bp-long genome containing 44 protein-coding genes and a G+C content of 30.4%. SerPounce is similar to the previously identified *Bacillus* phages Stitch (NCBI RefSeq no. NC_031032.1), Aurora (RefSeq no. NC_031121.1), MG-B1 (RefSeq no. NC_021336.1), and QCM11 (GenBank accession no. KX961631.1), with an ANI of 90% ± 3%. Phylogenetic analysis based on the presence/absence of phamilies provides strong support for a distinct clade encompassing Harambe and BeachBum, suggesting that they may define a new subcluster (5). In addition, a homolog of phi29 pRNA was detected in SerPounce but not in Harambe or BeachBum, consistent with extensive substantial rearrangement of structural genes in the genomes of these two phages (6, 7).

The phages displayed differing abilities to lyse lawns of the 10 different *B. cereus* group species tested. BeachBum has one of the more narrow host ranges observed, with crude lysate able to clear spots only on lawns of *B. cereus* FDA5 (ATCC 10702), *B. thuringiensis* subsp. *israelensis* (NRRL B-4489), PS52A1, and DSM 350, and *Bacillus anthracis* delta Sterne. Harambe crude lysate was able to clear spots on lawns of all of the *B. cereus* group spp. other than *B. thuringiensis* subsp. *israelensis*, and it was also able to clear *B. thuringiensis* subsp. Al Hakam and *B. cereus* strains Frankland and Frankland (ATCC 14579) and Gibson 971. SerPounce demonstrated one of the largest putative host ranges of the 60 phages tested in the 2016 to 2017 course and was able to lyse 9 of the 10 *B. cereus* group hosts tested, including all of the strains lysed by Harambe and BeachBum, except for *B. thuringiensis* subsp. *israelensis*, and was among the few (13%) tested phages able to lyse *B. thuringiensis* subsp. *konkukian* (NRRL B-23144).

### Accession number(s).

The complete genome sequences of *Bacillus* phages Harambe, BeachBum, and SerPounce are available in GenBank with the accession numbers KY821088, KY921761, and KY947509, respectively.
